# Studies of a genetic variant in *HK1 *in relation to quantitative metabolic traits and to the prevalence of type 2 diabetes

**DOI:** 10.1186/1471-2350-12-99

**Published:** 2011-07-25

**Authors:** Anette P Gjesing, Aneta A Nielsen, Ivan Brandslund, Cramer Christensen, Anneli Sandbæk, Torben Jørgensen, Daniel Witte, Amélie Bonnefond, Phillippe Froguel, Torben Hansen, Oluf Pedersen

**Affiliations:** 1 The Novo Nordisk Foundation Center for Basic Metabolic Research, Faculty of Health Sciences, University of Copenhagen, Universitetsparken 1-3, 2100 Copenhagen, Denmark; 2 Department of Clinical Biochemistry, Vejle Hospital, Kabbeltoft 25, 7100 Vejle, Denmark; 3 Institute of Regional Health Research, University of Southern Denmark, J.B. Winsloews Vej 9B, 5000 Odense, Denmark; 4 Department of Internal Medicine and Endocrinology, Vejle Hospital, Kabbeltoft 25, 7100 Vejle, Denmark; 5 Department of General Practice, University of Aarhus, Vennelyst Boulevard 6, 8000 Aarhus, Denmark; 6 Research Centre for Prevention and Health, Glostrup University Hospital, Nordre Ringvej, 2600 Glostrup, Denmark; 7 Faculty of Health Science, University of Copenhagen, Blegdamsvej, 2200 Copenhagen, Denmark; 8 Steno Diabetes Center, Niels Steensens Vej 2, 2800 Gentofte, Denmark; 9 CNRS-UMR-8199, Lille Pasteur Institute, Univ Lille Nord de France, rue du Pr. Calmette, 59000 Lille, France; 10 Department of Genomics of Common Disease, School of Public Health, Imperial College London, Hammersmith Hospital, Du Cane Rd., London W12 0NN, UK; 11 Faculty of Health Sciences, University of Southern Denmark, J.B. Winsloews Vej 19, 5000 Odense, Denmark; 12 Hagedorn Research Institute, Niels Steensens Vej 1, 2820 Gentofte, Denmark; 13 Institute of Biomedical Science, Faculty of Health Sciences, University of Copenhagen, Blegdamsvej 3, 2200 Copenhagen, Denmark; 14 Faculty of Health Sciences, University of Aarhus, Aarhus, Denmark

**Keywords:** Hexokinase 1, Glycated Haemoglobin A1c, Type 2 diabetes, Genetics

## Abstract

**Background:**

Single nucleotide polymorphisms (SNPs) within the gene encoding Hexokinase 1 (*HK1) *are associated with changes in glycated haemoglobin (HbA1c) levels. Our aim was to investigate the effect of *HK1 *rs7072268 on measures of glucose- and lipid-metabolism in a Danish non-diabetic population and combine the outcome of these analyses in a meta-analysis with previously published results. Furthermore, our aim was to perform a type 2 diabetes case-control analysis and meta-analysis with two previous case-control studies.

**Methods:**

SNP rs7072268 was genotyped in 9,724 Danes. The quantitative trait study included 5,604 non-diabetic individuals from the Inter99 cohort. The case-control study included 4,449 glucose tolerant individuals and 3,398 patients with type 2 diabetes. Meta-analyses on quantitative traits included 24,560 Caucasian individuals and 30,802 individuals were included in the combined analysis of present and previous type 2 diabetes case-control studies.

**Results:**

Using an additive model, we confirmed that the T-allele of rs7072268 associates with increased HbA1c of 0.6% (CI: 0.4 - 0.9), *p *= 3*10^-7 ^per allele. The same allele associated with an increased area under the curve (AUC) for glucose of 5.0 mmol/l*min (0.1 - 10.0), *p *= 0.045 following an oral glucose tolerance test (OGTT) and increased fasting levels of cholesterol of 0.06 mmol/l (0.03 - 1.0), *p *= 0.001 and triglycerides of 2.0% (0.2 - 3.8), *p *= 0.03 per allele in the same study sample of non-diabetic individuals from the Inter99 cohort. However, the T-allele did not show any association with estimates of insulin release or insulin sensitivity neither in Inter99 nor in combined analyses. The prevalence of type 2 diabetes was increased among carriers of the rs7072268 T-allele both in the Danish study-population with an OR of 1.11 (1.02-1.21) and in a meta-analysis including the two additional sample sets with an OR of 1.06 (1.02-1.11). However, after Bonferroni correction the T-allele only remained associated to HbA1c and fasting cholesterol.

**Conclusions:**

The present study provides suggestive evidence of an association of the rs7072268 T-allele in *HK1 *with increased AUC glucose following an OGTT in non-diabetic individuals and a nominal association with type 2 diabetes prior to Bonferroni correction. The latter was confirmed in combined analyses involving 16,445 cases and 14,357 control subjects.

## Background

The T-allele of rs7072268, located in intron 7 of the gene encoding the hexokinase 1 (*HK1*), has previously been associated with higher glycated haemoglobin levels (HbA1c) in a genome-wide association study (GWAS) among more than 14,000 women [1]. These findings were confirmed in another study showing an association between the same SNP allele and increased HbA1c levels [2]. A different common variant located in *HK1 *(rs16926246) has also been identified in a GWAS as a variant associating with increased HbA1c levels [3]. Yet, rs7072268 was further investigated for association with glucose homeostasis-related traits such as fasting glucose, fasting insulin, HOMA-B (Homeostatic model assessment of β-cell function) and HOMA-IR (Homeostatic model assessment of insulin resistance) but no significant effects were found [2]. However, it has been shown that rs7072268 associates with red blood cells - variables, e.g. red blood cell count, hematocrit, and anemia and not to type 2 diabetes (T2DM) related traits [2].

*HK*1 is expressed in all mammalian tissues, and is considered a "housekeeping enzyme" [4]. HK1 is responsible for the first step in glucose utilization and it has been hypothesized that *HK1 *variants may affect glucose metabolism and thereby risk of T2DM [1]. Thus, the aim of present study was to validate the previously observed outcome of the rs7072268 within *HK1 *on 1) quantitative metabolic traits in a random sample of 5,604 adult Danish non-diabetic individuals with available data on glucose homeostasis and lipid variables and 2) on prevalence of T2DM in a case-control study including 4,449 glucose tolerant individuals and 3,398 patients with T2DM. Furthermore, we combined the outcomes of the present analyses with previously published results in a meta-analysis.

## Methods

### Danish study populations

Individuals included in the present study were ascertained from three study populations: 1) 6,282 individuals were included from a population-based, randomized, non-pharmacological intervention study for the prevention of ischemic heart disease including middle-aged individuals; Inter99 (http://ClinicalTrials.gov identification no. NCT00289237) [5]. 2) 1,621 individuals from a population-based, high-risk screening and intervention study for T2DM in general practice; ADDITION (Anglo-Danish-Dutch Study of Intensive Treatment in People with Screen-Detected Diabetes in Primary Care) (http://ClinicalTrials.gov ID-no: NCT00237548) [6]. 3) 1,439 individuals from a sample of unrelated type 2 diabetic patients sampled through the out-patient clinic at Steno Diabetes Center; SDC. Among Inter99 participants glucose tolerance status was defined according to WHO 1999 criteria [7] and in the present quantitative trait study we included only non-diabetic individuals from Inter99.

The case-control study included 4,449 glucose tolerant individuals and 3,398 patients with T2DM. For further description see Table 1. Patients having diabetes as a consequence of known chronic pancreatitis, haemochromatosis, mutations in the insulin receptor, lipodystrophy, maturity-onset diabetes of the young, maternally inherited diabetes and deafness, family history of first-degree relatives with Type 1 diabetes, insulin requirement within the first year after diabetes diagnosis or a fasting serum C-peptide level ≤ 150 pmol/l at time of recruitment were excluded from the category of clinically defined T2DM.

**Table 1:** Description of study samples included in the case-control study

Trait	Inter99:	Inter99:	Addition:	SDC:
Glucose tolerance status	NGT	T2DM	T2DM	T2DM

N (men/women)	4449 (2062/2379)	343 (197/126)	1613 (918/695)	1439 (902/537)

Mean age (years)	45.2 ± 7.8	51.6 ± 7.9	60.3 ± 6.8	63.1 ± 11.0

Mean BMI (kg/m^2^)	25.5 ± 4.1	30.3 ± 5.8	31.1 ± 5.4	30.5 ± 5.6

Data are presented as mean ± SD. NGT, normal glucose tolerance; T2DM, Type 2 diabetes patients.

All participants were Danish Caucasians by self-report, and informed written consent was obtained from all subjects before participation. The studies were approved by the Ethical Committees of Copenhagen and Aarhus, and were in accordance with the principles of the Helsinki Declaration II.

### Study samples included in the combined analyses

Details of the French study populations included in the meta-analyses are reported by Bonnefond and colleagues [2]. In short, five Caucasian study populations including a total of 18,956 non-diabetic individuals were included in the study. This study also included a case-control study conducted among 7,447 patients with T2DM and 5,380 normoglycemic participants [2].

Participants from the Diabetes Genetics Replication And Meta-analysis (DIAGRAM) case-control study included 4,549 type 2 diabetic patients and 5,579 control individuals. These individuals were collected from three previous T2DM GWAS. Participants were of Caucasian descent and are further described by Zeggini and colleagues [8].

### Anthropometrics and biochemical assays

Height and weight were measured in light indoor clothes and without shoes, and BMI was calculated as weight (kg)/height (m)^2^. Waist circumference at the umbilical level was measured on subjects in an upright position to the nearest 0.5 cm using a non-extendable linen tape measure according to WHO recommendation.

Blood samples were drawn after a 12-h overnight fast. Plasma glucose was in the Danish study par-ticipants analysed by a glucose oxidase method (Granutest, Merck, Darmstadt, Germany), and serum insulin (excluding des (31,32) and intact proinsulin) was measured using the AutoDELFIA insulin kit (Perkin-Elmer, Wallac, Turku, Finland). Serum triglycerides and total and HDL-serum cholesterol were analysed using enzymatic colorimetric methods (GPO-PAP and CHOD-PAP, Roche Molecular Biochemicals, Germany). HbA1C was measured using ion-exchange high performance liquid chromatography (normal reference range: 4.1-6.4%).

### Genotyping

Genotyping of the *HK1 *rs7072268 was in the Danish study sample performed using a Sequenom iPlex assay [9]. The genotyping success rate was 98% and the error rate was 0% in 203 duplicate samples. All groups of genotypes obeyed Hardy-Weinberg equilibrium.

### Statistical analysis

Quantitative trait analysis was performed in the Inter99 study population and did not include patients with screen-detected or known diabetes. A general linear model was used to test quantitative variables (or transformed variables) for differences between genotype groups. Genotype and sex were considered as fixed factors and age and BMI as covariates. Traits not following a normal distribution were log-transformed prior to analysis. To examine genotype distribution differences between affected and unaffected subjects logistic regression analysis was applied including adjustment for sex, age and BMI. Individuals with unknown diabetes status were excluded from case-control analyses for T2DM. The meta-analyses were performed using effect size estimates and SE derived from a linear regression analysis for quantitative traits and OR with CI for case-control studies. The effects for quantitative traits were based on log transformed traits except for values of glucose. In the meta-analyses both fixed effect (weight of studies estimated using inverse variance) and random effect (weight of studies estimated using DerSimonian-Laird method) [10] were applied. All studies were adjusted for age, sex and BMI. All analyses were performed using RGui version 2.7.0. A two-sided p-value of less than 0.05 was considered to be significant.

### Indices

Insulinogenic index: ((serum insulin 30 min - fasting serum insulin))/(fasting plasma glucose)

HOMA-B: (20 * fasting serum insulin)/(fasting plasma glucose - 3.5)

HOMA-IR: (fasting plasma glucose * fasting serum insulin)/22.5

ISI: 10000/√(((fasting plasma glucose (mmpl/l) * 18) * (fasting serum insulin (pmol/l)/6) * (mean glucose (mmol/l) * 18) * (mean insulin (pmol/l)/6)))

Disposition index: ISI * Insulinogenic index

## Results

Using an additive model adjusted for age, sex and BMI, the impact of the T-allele of rs7072268 in *HK1 *on quantitative metabolic variables was investigated in non-diabetic adults from the Inter99 cohort. The T-allele associated with an increase in HbA1c of 0.6% per allele (0.4 - 0.9); p = 3 × 10^-7^, an increase of 5.0 mmol/l×min per allele (0.1 - 10.0); p = 0.045 for AUC for glucose under an OGTT, an increase of 0.06 mmol/l per allele (0.03 - 1.0); p = 0.001 in fasting total plasma cholesterol, and an increase of 2.0% per allele (0.2 - 3.8); p = 0.03 in fasting plasma triglycerides (Table 2). However, only associations with HbA1c and total plasma cholesterol remained significant following Bonferroni correction for multiple testing (Table 2).

**Table 2:** Quantitative trait analyses of the effect of rs7072268 *of HK1 *among 5,604 non-diabetic individuals from the Inter99 study population

Trait	CC	CT	TT	P_add_	B_add_ (CI)
N (men/women)	1545 (765/780)	2757 (1347/1410)	1302 (644/658)		

Age (years)	46.1 ± 8.0	45.8 ± 7.9	45.9 ± 7.7		

**Body composition**					

BMI (kg/m^2^)	25.9 ± 4.4	26.1 ± 4.4	26.0 ± 4.3	0.5	0.06 (-0.10; 0.22)

Waist-to-hip ratio	0.85 ± 0.08	0.85 ± 0.09	0.85 ± 0.09	0.2	0.001 (-0.001; 0.003)

Waist (cm)	86.7 ± 12.9	86.1 ± 12.8	85.8 ± 12.8	0.9	-0.12 (-0.20; 0.18)

**Lipids**					

Triglycerides* (mmol/l)	1.0 (0.8; 1.4)	1.1 (0.8; 1.5)	1.1 (0.8; 1.5)	0.03	2.0 (0.2;3.8)

Cholesterol (mmol/l)	5.5 ± 1.0	5.5 ± 1.1	5.6 ± 1.1	0.001	0.06 (0.03; 1.0)

HDL cholesterol (mmol/l)	1.4 ± 0.4	1.4 ± 0.4	1.4 ± 0.4	0.7	0.003 (-0.010; 0.016)

LDL cholesterol (mmol/l)	3.5 ± 1.0	3.5 ± 0.9	3.6 ± 1.0	0.1	0.04 (-0.01; 0.09)

VLDL cholesterol (mmol/l)	0.6 ± 0.3	0.6 ± 0.3	0.6 ± 0.3	0.1	0.01 (-0.004; 0.03)

**Serum Insulin**					

Fasting serum Insulin * (pmol/l)	33.0 (23.0; 47.0)	34.0 (24.0; 51.0)	33.0 (23.0; 51.0)	0.2	1.2 (-0.7; 2.7)

Serum insulin 30 min * (pmol/l)	242 (173; 355)	250 (179; 354)	244 (179; 359)	0.6	0.5 (-1.4; 2.4)

Serum insulin 120 min * (pmol/l)	151 (94.3; 242)	155 (96.0; 249)	148 (91.0; 248)	0.9	0.1 (-2.6; 2.9)

AUC insulin* (pmol/l * min)	22460 (16200; 32620)	23490 (16950; 32770)	22820 (16770; 32910)	0.2	0.8 (-1.1; 2.6)

**Plasma glucose**					

Fasting Plasma glucose (mmol/l)	5.4 ± 0.5	5.5 ± 0.5	5.5 ± 0.5	0.2	0.01 (-0.004; 0.03)

Plasma glucose 30 min (mmol/l)	8.5 ± 1.67	8.6 ± 1.7	8.6 ± 1.7	0.1	0.05 (-0.004; 0.1)

Plasma glucose 120 min (mmol/l)	5.9 ± 1.56	6.0 ± 1.5	6.0 ± 1.5	0.1	0.04 (-0.02; 0.09)

AUC glucose (mmol/l* min)	856 ± 143	865 ± 145	866 ± 144	0.045	5.0 (0.1; 10.0)

**Indices of insulin sensitivity**					

HOMA-IR*	7.8 (5.4; 11.6)	8.3 (5.7; 12.6)	7.9 (5.4; 12.8)	0.2	1.3 (-0.6; 3.3)

DI*	204 (140; 291)	194 (130; 279)	197 (129; 285)	0.3	-1.2 (-3.3; 0.9)

ISI*	8.3 (5.6; 9.5)	7.8 (5.3; 11.1)	8.0 (5.2; 11.6)	0.1	-1.4 (-3.2-0.4)

**Indices of acute insulin response**					

Insulinolinogenic Index*	24.9 (17.3; 37.1)	25.1 (17.0; 37.3)	24.7 (17.4; 36.1)	0.8	0.4 (-1.8; 2.5)

**Glycated haemoglobin levels**					

Hba1c* (%)	5.8 (5.5; 6.0)	5.8 (5.5; 6.1)	5.8 (5.6; 6.1)	3e-07	0.6 (0.4; 0.9)

Data are presented as mean ± SD and as effect sizes (95% CI) for traits following a normal distribution. Remaining traits are presented as median (inter-quartile range) and their effect sizes are presented as increase/decrease in percentage. Multiple regression analysis was used to test for difference between genotype groups. P-values are corrected for sex and age. * = log transformation.

**Figure 1:**
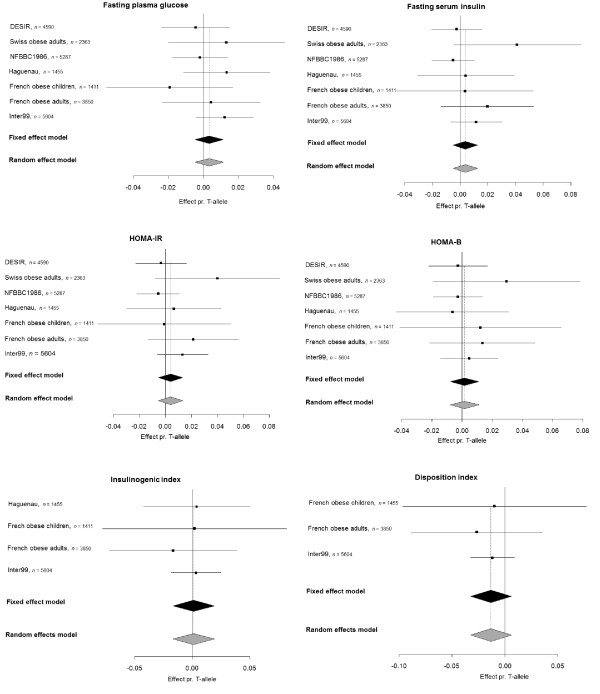
**Meta-analyses estimating the combined effect and 95% confidence interval of the T-allele of rs7072268 in *HK1 *from the present study and the study by Bonnefond et al**. [2]**on quantitative metabolic traits**. Estimates for fasting insulin, HOMA-IR, HOMA-B, Insulinogenic index and disposition index are based on log transformed traits. The black diamonds represent the combined effects of the studies weight using inverse variance. The grey diamonds represent the combined effects of the studies which were weighted using the DerSimonian-Laird method.

To increase the statistical power of the study, we performed a meta-analysis including quantitative metabolic traits data from a previously published French study including 18,956 Caucasian participants [2]. The traits investigated in this study included the following: fasting plasma glucose, fasting serum insulin, HOMA-B, HOMA-IR, plasma glucose 30 min post-OGTT, plasma glucose 120 min post-OGTT, serum insulin 30 min post-OGTT, serum insulin 120 min post-OGTT, insulinogenic index, diposition index, index of insulin sensitivity and AUC for glucose. None of these combined analyses revealed any significant effect of the *HK1 *variant on traits related to glucose homeostasis (Figure 1 and Additional file 1, Figure S1).

**Figure 2:**
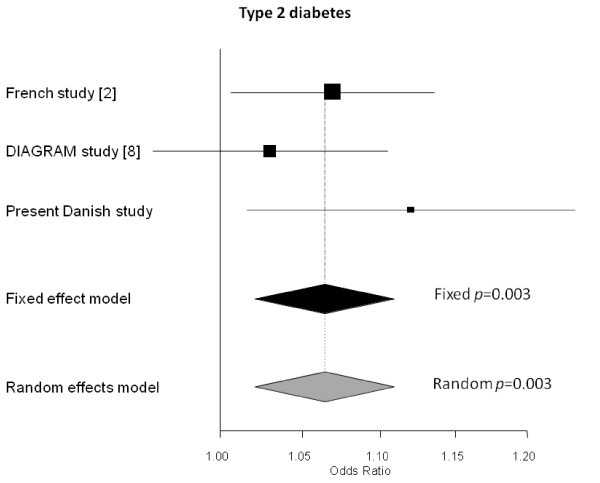
**Meta-analyses estimating the combined effect and 95% confidence interval of the T-allele of rs7072268 in *HK1 *from the present study, the study by Bonnefond et al**. [2]** and the DIAGRAM study **[8]** on risk of type 2 diabetes**. The black diamonds represent the combined effects of the studies weight using inverse variance. The grey diamonds represent the combined effects of the studies which were weighted using the DerSimonian-Laird method.

In a case-control setting we examined the impact of rs7072268 of *HK1 *on prevalence of T2DM. Logistic regression analysis revealed an association between the T-allele of rs7072268 and T2DM among 4,449 glucose tolerant individuals and 3,389 T2DM patients with an OR of 1.11 (1.02-1.21), p = 0.02 (Table 3). The outcome of the Danish case-control study was included in a meta-analysis involving 7,447 French T2DM cases and 5,380 French control subjects [2] as well as 4,549 T2DM cases and 5,579 control subjects from the DIAGRAM consortium [8]. This meta-analyses also found a significant association between the T-allele of rs7072268 and T2DM with an OR of 1.064 (1.021-1.11), *p *= 0.003 (Figure 2).

**Table 3:** Estimation of the effect of rs7072268 of *HK1 *on the risk of T2DM in a case-control study among 9,724 Danes

	NGT	T2DM	OR (95% CI) 1	OR (95% CI) 2	P1	P2
CC (%)	1244(28)	938(27.7)				

CT (%)	2182 (49)	1686 (49.7)				

TT (%)	1023 (23)	765 (22.6)				

MAF (95% CI)	47.5 (46.5-48.6)	47.4 (46.3-48.6)	1.11 (1.02-1.21)	1.12 (1.02-1.24)	0.02	0.02

OR (95% CI) 1; Odd ratio adjusted sex and age. OR (95% CI) 2; Odd ratio adjusted sex, age and BMI. P-values are calculated using logistic regression. P1; Adjusted for sex and age. P2; Adjusted for sex, age and BMI. T2DM; Type 2 diabetes, NGT; Normal glucose tolerance.

## Discussion

In line with a previous study, we found that the T-allele of rs7072268 in *HK1 *was associated with an increased concentration of HbA1c in a Danish non-diabetic population. This effect of rs7072268 is comparable to the results obtained from another independent variant located in *HK1 *(rs16926246) which also associated with measures of HbA1c when analysing 46,368 nondiabetic European individuals [3]. These two variants are in low linkage disequilibrium (r^2 ^= 0.1). We also found an increased level of AUC for glucose following oral glucose ingestion and increased measures of fasting circulating levels of triglyceride and total cholesterol. However, the remaining traits related to glucose homeostasis following oral glucose ingestion failed to reveal any association with the rs7072268 variant in *HK1*. A French study also failed to identify any significant associations between the rs7072268 and glucose regulation-related traits. We investigated these previous traits further in a meta-analysis including a total of 24,560 individuals; yet, no significant associations were found. However, lipid-related measures were not among the previously examined traits and the significant associations found in the Danish study could therefore not be further validated in a meta-analysis. *HK1 *was not identified as a locus associating with fasting glucose, fasting insulin, HOMA-B and HOMA-IR in a meta-analysis of 21 GWAS [11].

Another significant finding was the association between the T-allele of rs7072268 and T2DM. This finding was further supported by a meta-analysis combining the effect of the rs7072268 variant in this study with two additional studies [2,8]. This effect was not significant after Bonferroni correction. However, Bonferroni correction is a very conservative method assuming independence between traits which is not a correct assumption for the included traits and validation of the present result in an independent study sample is crucial. However, the largest type 2 diabetes GWAS conducted to date failed to identify a genome-wide significant effect of this variant [12]. Nevertheless, the high threshold of significance for GWAS may cause true associations having only minor effects to be hidden in the fog of random associations and the effect of rs7072268 on type 2 diabetes observed in present study may not be a result of a type 1 error.

Biologically, hexokinase 1 is one of the key enzymes of glycolysis and catalyzes the phosphorylation of glucose to glucose-6-phosphate on the mitochondria. HK1 is found in all mammalian tissues; yet, the only tissues depending solely on HK1 for glucose utilisation are tissues having a strict dependence on glucose utilization for their physiologic functions, such as brain, erythrocytes, platelets, lymphocytes, and fibroblasts [13-15]. A minor defect in *HK1 *may therefore mainly be apparent intracellular in such tissue without causing measureable physiological changes such as circulating plasma glucose, as the majority of tissue is relaying on several hexokinases, possibly compensating for such a minor defect. Therefore, despite the lack of significant effects on measures of circulating glucose, rs7072268 may have an effect on intracellular glycemic metabolism, which is possibly causing the association with increased HbA1c.

There is no direct biological link between the function of HK1 and circulating cholesterol levels and the observed association may despite Bonferroni correction be a type 1 error thus replication of this finding is essential. However, the association with increased levels of fasting lipids observed in non-diabetic individuals may be an early metabolic consequence of this variant related to a slightly altered glucose metabolism, as it is well known that adverse changes in lipids - such as increased triglyceride and cholesterol levels - are seen in pre-diabetic and/or metabolic syndrome individuals long before the onset of T2DM [16]. Therefore, it is plausible that rs7072268 or a functional variant within close proximity has an effect on intracellular glucose utilisation which may indirectly affect lipid metabolism.

A monogenic form of HK1 deficiency has been described and the primary effect of this deficiency was hemolytic anemia, however, there were no information regarding the glycemic control of these patients [17]. Also, rs7072268 has previously been associated with a pro-anemic state [2]. However, whether there is a connection between glucose-metabolism and anemia is not well established. It has recently been found that the frequency of anemia was increased in diabetic patients [18] and it was seen in a Chinese population that anaemia was associated with an increased risk of hyperglycaemia [19]. Thus, it is possible that HK1 may be a factor influencing both maintenance of the red blood cells pool and glucose-homeostasis and may even be a link between them.

Despite the large number of individuals included, the present may be underpowered as the effects of the rs7072268 on measure of glucose homeostasis are minor, thus requiring a large number of participants.

## Conclusion

Based on the present results, we suggest that the T-allele of rs7072268 in *HK1 *associates with T2DM as well as fasting dyslipidaemia in non-diabetic individuals prior to Bonferroni correction.

## Abbreviations

AUC: Area Under the Curve; GWAS: Genome Wide Association Study; HbA1c: Glycated Haemoglobin 1c; HK: Hexokinase; ISI: Insulin Sensitivity Index; SNP: Single Nucleotide Polymorphism; T2DM: Type 2 Diabetes.

## Competing interests

The authors declare that they have no competing interests.

## Authors' contributions

APG and AAN were involved the generation of the original hypothesis and in the analyses, the in-terpretation of results and the drafting of the manuscript. IB and CC was involved in the supervision of the study regarding hypothesis generation, analysis and interpretation of results. TJ, DW, TH, IB, CC, AAN and OP were involved in the initiation and collection of the Danish study population. PF was involved in the initiation and collection of the French study populations. AB generated data included in the meta-analyses. TH and OP conceived the study, and participated in its design and coordination and helped to draft the manuscript. All authors read and approved the final manuscript.

## Pre-publication history

The pre-publication history for this paper can be accessed here:

http://www.biomedcentral.com/1471-2350/12/99/prepub

## Supplementary Material

Additional file 1 **Figure S1**. Meta-analyses estimating the combined effect and 95% confidence interval of the T-allele of rs7072268 in *HK1 *from from the present study and the study by Bonnefond and co-workers [2] on plasma glucose 30 and 120 minutes after an OGTT, serum insulin 30 and 120 minutes after an OGTT and insulin sensitivity index (ISI). **Below Figure S1: **Estimates for insulin and insulin sensitivity index (ISI) are based on log transformed traits. The black diamonds represent the combined effects of the studies weight using inverse variance. The grey diamonds represent the combined effects of the studies which were weighted using the DerSimonian-Laird method. Click here for file
