# Supplementary figures and images for: Studies of a genetic variant in HK1 in relation to quantitative metabolic traits and to the prevalence of type 2 diabetes
Source: BMC Med Genet. 2011 Jul 25;12:99. doi: 10.1186/1471-2350-12-99 (PMC3161933; doi:10.1186/1471-2350-12-99)

Plasma glucose 30 min

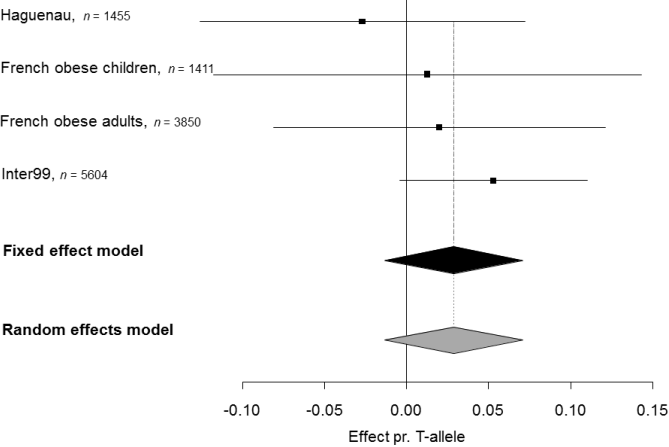

Plasma glucose 120 min

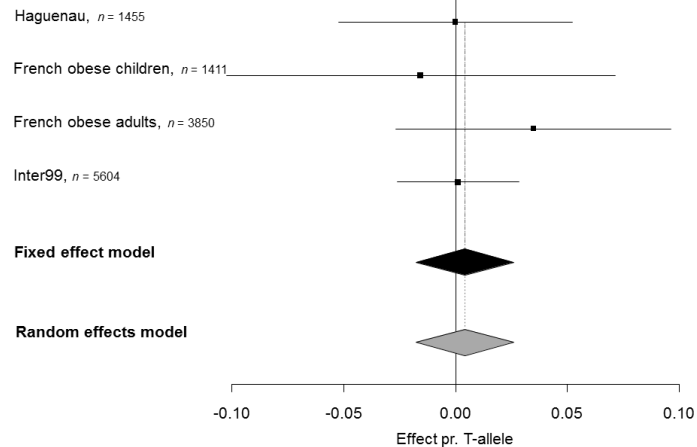

Serum insulin 30 min

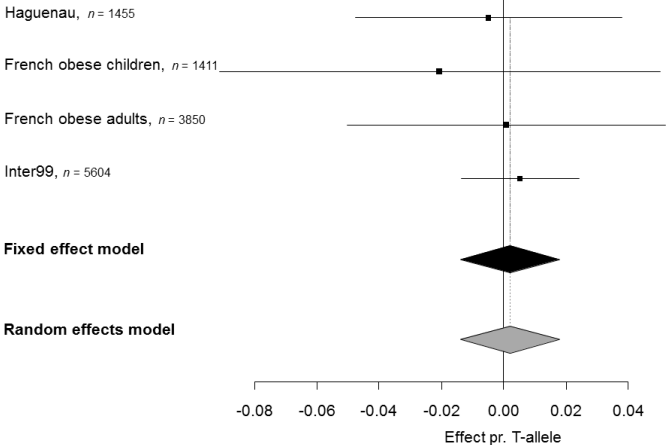

Serum insulin 120 min

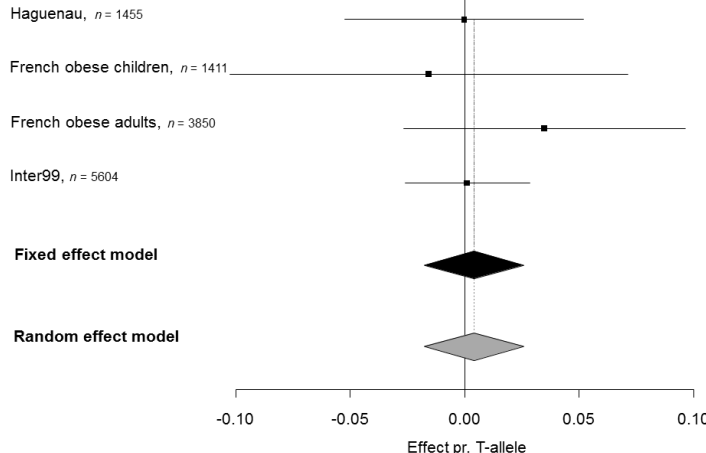

ISI

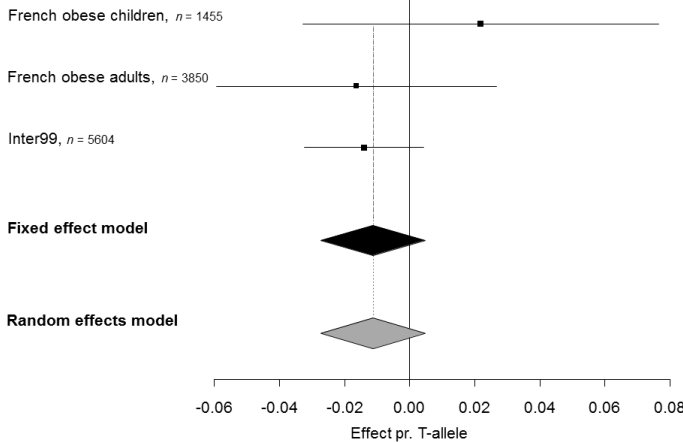

AUC glucose

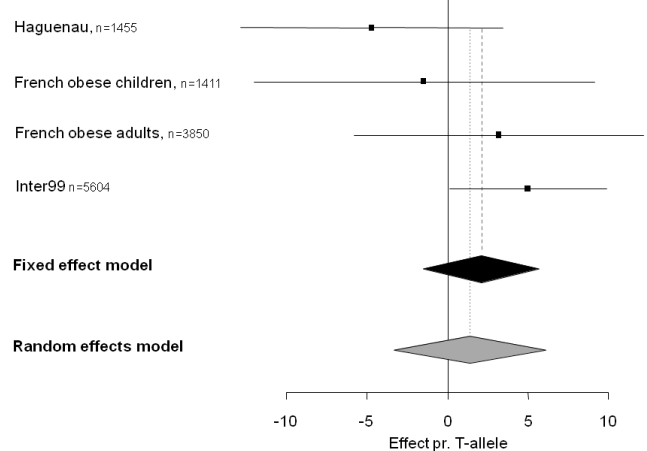

Supplement: Additional file 1 — Figure S1. Meta-analyses estimating the combined effect and 95% confidence interval of the T-allele of rs7072268 in HK1 from from the present study and the study by Bonnefond and co-workers [2] on plasma glucose 30 and 120 minutes after an OGTT, serum insulin 30 and 120 minutes after an OGTT and insulin sensitivity index (ISI). Below Figure S1: Estimates for insulin and insulin sensitivity index (ISI) are based on log transformed traits. The black diamonds represent the combined effects of the studies weight using inverse variance. The grey diamonds represent the combined effects of the studies which were weighted using the DerSimonian-Laird method. [file 1471-2350-12-99-S1.PDF]
